# Death Registration in Texas

**Published:** 1911-05

**Authors:** 


### Death Registration in Texas.

A NUMBER OF CITIES MAY BE ADMITTED TO CENSUS BUREAU'S AREA THIS YEAR.

Although Texas, as a State, is not included in the death registration area of the United States formed for the compilation and study of mortality statistics by the Bureau of Census, there are two cities, Galveston and San Antonio which, because of effective local death registration ordinances, have been since 1906 comprised in the Bureau's area.

Owing to the activity of Dr. William M. Brumby, the former State Health Officer, in promoting the extension of the registration area, many requests from Texas cities which desire admission to the area have been received by the Census Bureau. These will be carefully considered and Dr. Cressy L. Wilbur, Chief Statistician for Vital Statistics in the Bureau, states that it is probable that a considerable number of them, in which the ordinances are thoroughly enforced, may be admitted for the current year 1911.

---

DR. GEO. L. SERVOSS has removed from Fairview, Nevada, to Fallon, Nevada.

